# North Midland Poor-Law Conference

**Published:** 1910-10-29

**Authors:** 


### NORTH MIDLAND POOR-LAW CONFERENCE.

The twenty-second annual meeting of the above Conference for the counties of Derby, Leicester, Lincoln, Nottingham, and Rutland was opened in the Leicester Town Hall, thirty-one Unions being represented. The Mayor of Leicester (Councillor Chitham) presided, and gave a hearty welcome to the delegates to the county town. The Hon. Secretary (Mr. James Blossom) referred to the list of the Unions represented, and pointed out that the total number of names returned was 122, a larger number by far than had attended any previous Conference. The Mayor of Leicester having to leave, the chair was taken by Mr. Thos. Palmer (Nottingham), Chairman of the Conference Committee.

Mr. J. L. Harrison, a member of the Leicester Board of Guardians, then read a paper on "Co-operation between Charity and the Poor Law—a plea for its more general application," in which he urged that the ultimate responsibility for the welfare of every person relieved should be vested in the Guardians, and that charity should not prevent a person applying for relief to which his need rendered him legally entitled. He suggested that children should be provided for in cottage homes or scattered homes under the control of the Guardians, but that steps should be taken to provide effective treatment in certificated schools for the blind, deaf, dumb, and cripples. The Guardians had power to avail themselves of the advantages of such homes, which he recommended should be used to the full. The cost of this system was considerably less than any under which the Guardians themselves built the necessary homes, and the children secured the specialised treatment they would not otherwise receive. The after-care of cases so treated should be systematically provided for, and they could with great advantage avail themselves of the services of a number of voluntary lady workers. With regard to the aged and incurable, many could be looked after in less costly institutions than the modern Poor-Law hospital, and the Guardians, therefore, should co-operate with the voluntary agencies in placing these persons in suitable surroundings. The keynote in dealing with old people needing relief should be consideration for their feelings. Under whatever scheme the Poor Law was to be administered in the future, the best results could only be obtained from intelligent and organised co-operation between charity and the local authority.

Discussion on the paper followed, subsequent to which Col. Gerard Clark (Hon. Secretary to the Central Committee of Poor-Law Conferences and a Guardian of Paddington) read a paper on "The Remoulding of Poor-Law Administration, with special reference to Mr. Charles Booth's memoranda." In the evening a large number of the delegates assembled at dinner at the Grand Hotel, Mr. Joseph Howe (Chairman of the Leicester Guardians) presiding.

